# Performance comparison of TCR-pMHC prediction tools reveals a strong data dependency

**DOI:** 10.3389/fimmu.2023.1128326

**Published:** 2023-04-18

**Authors:** Lihua Deng, Cedric Ly, Sina Abdollahi, Yu Zhao, Immo Prinz, Stefan Bonn

**Affiliations:** ^1^ Institute of Systems Immunology, University Medical Center Hamburg-Eppendorf, Hamburg, Germany; ^2^ Institut of Medical Systems Biology, University Medical Center Hamburg-Eppendorf, Hamburg, Germany

**Keywords:** T-cell receptor (TCR), peptide, MHC, machine learning/deep learning, TCR specificity prediction

## Abstract

The interaction of T-cell receptors with peptide-major histocompatibility complex molecules (TCR-pMHC) plays a crucial role in adaptive immune responses. Currently there are various models aiming at predicting TCR-pMHC binding, while a standard dataset and procedure to compare the performance of these approaches is still missing. In this work we provide a general method for data collection, preprocessing, splitting and generation of negative examples, as well as comprehensive datasets to compare TCR-pMHC prediction models. We collected, harmonized, and merged all the major publicly available TCR-pMHC binding data and compared the performance of five state-of-the-art deep learning models (TITAN, NetTCR-2.0, ERGO, DLpTCR and ImRex) using this data. Our performance evaluation focuses on two scenarios: 1) different splitting methods for generating training and testing data to assess model generalization and 2) different data versions that vary in size and peptide imbalance to assess model robustness. Our results indicate that the five contemporary models do not generalize to peptides that have not been in the training set. We can also show that model performance is strongly dependent on the data balance and size, which indicates a relatively low model robustness. These results suggest that TCR-pMHC binding prediction remains highly challenging and requires further high quality data and novel algorithmic approaches.

## 1 Introduction

T-cell receptors (TCR) play a crucial role in adaptive immunity mainly through the recognition of peptide fragments from foreign pathogens that are presented by major histocompatibility complex (MHC) molecules. TCRs consist of two transmembrane polypeptide chains, α and β chain; they form a heterodimer on the cell surface. The extraordinary diversity of the TCR repertoire is mainly attributed to a somatic recombination process, V(D)J recombination. Humans can theoretically generate more than 10^15^ different antigen-specific TCRs Uziela et al. (1). The diversity of TCR α and β is realized mainly by the complementarity-determining regions (CDRs), with CDR3 being the contact side to the peptide fragment and consequently the most important area for antigen recognition Hennecke and Wiley (2). There are two types of MHC molecules, MHC class I and MHC class II molecules, presenting peptides to CD8^+^ and CD4^+^ T cells, respectively.

The major public data resources for TCR-pMHC binding data are VDJdb Goncharov et al. (3), IEDB Vita et al. (4), McPAS-TCR Tickotsky et al. (5), ImmuneCODE Nolan et al. (6), TBAdb Zhang et al. (7) and 10X Genomics 10x Genomics (8), which all contain TCR CDR3 β chain information. These are all precious data since identifying cognate TCRs-pMHC binding pairs typically needs both the pMHC multimers technology and single cell sequencing technology Pai and Satpathy (9); Joglekar and Li (10).

This vast diversity of the TCR repertoire makes it difficult to experimentally cover all possible TCR pMHC binding pairs. Under the fundamental assumption that the binding between TCR and pMHC is governed by fundamental physicochemical interaction rules, computational approaches could detect and learn patterns in data. Applying machine learning (ML) and deep learning (DL) approaches to predict the interaction between TCR and pMHC have been explored, resulting in various models such as TITAN, NetTCR-2.0, ERGO, DLpTCR and ImRex Weber et al. (11); Montemurro et al. (12); Springer et al. (13); Xu et al. (14); Moris et al. (15). Among these models, ERGO and TITAN integrated natural language processing (NLP) techniques, NetTCR-2.0 and ImRex are based on convolutional neural networks (CNN), and DLpTCR is a combination of CNN, fully connected network (FCN) and deep residual network (ResNet). Unfortunately, to date there exists no appropriate benchmark dataset or workflow to compare contemporary TCR-pMHC prediction models and improve them. In this work, we collected and preprocessed all available major TCR-pMHC data and compared the performance of those state-of-the-art models in different training and testing scenarios.

## 2 Results

### 2.1 Current available data showed a great imbalance

**Figure 1:**
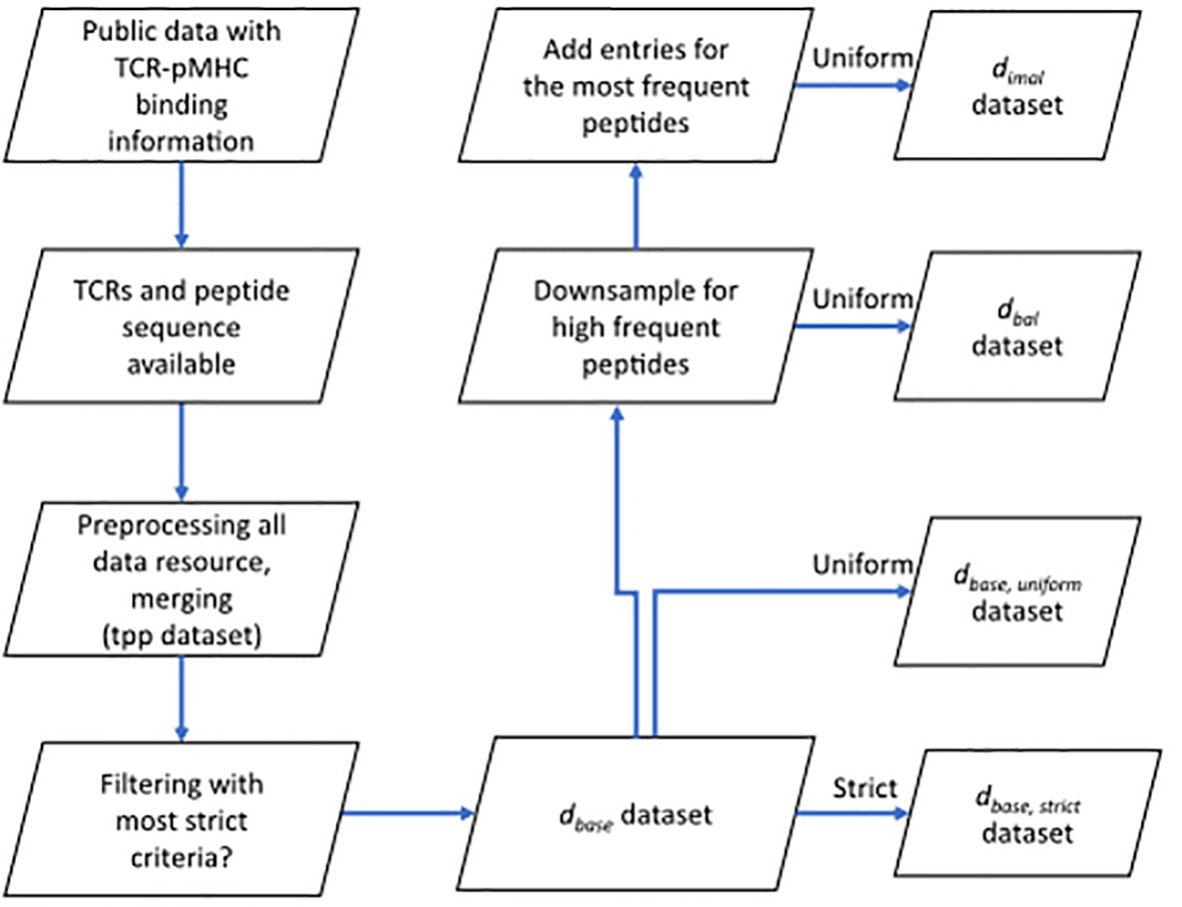
Flow chart shows the basic procedure for preparing different datasets. After collecting data from public resources and merging the preprocessed into one dataset (TCR preprocessed dataset, tpp dataset), different filtering criteria were applied to obtain the positive examples for $d_{base,strict}$ , $d_{base,uniform}$ , $d_{bal}$ and $d_{imbal}$ datasets. Negative examples were generated within folds (refer to 4.1.3) after splitting (refer to 4.1.2) to obtain the complete datasets. $d_{base}$ : the base dataset filtered from tpp dataset. $d_{base,strict}$ : strict splitting used on $d_{base}$ . $d_{base,uniform}$ : uniform splitting used on $d_{base}$ . $d_{bal}$ : the balanced dataset filtered from $d_{base}$ , then split using uniform splitting. $d_{imbal}$ : the imbalance dataset filtered from $d_{base}$ , then split using uniform splitting.

**Figure 2:**
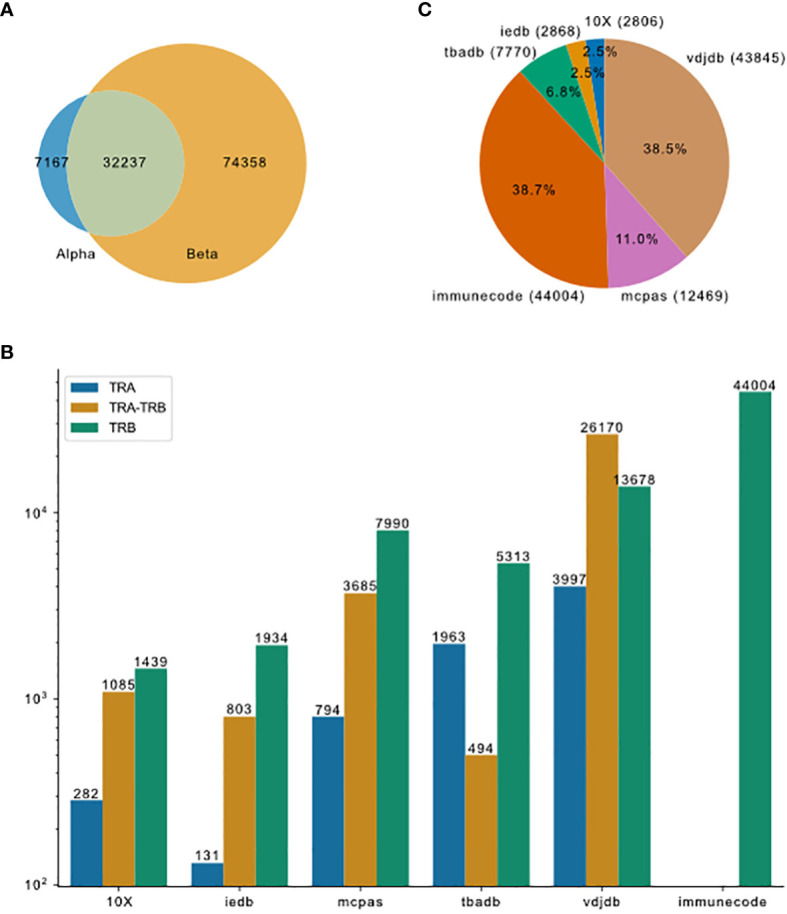
Overview of TCR-pMHC binding data merged from different resources. **(A)** Venn plot shows the overlap of entries that contain only TRA, paired chains or only TRB. The size of the ellipses correlate to the number of entries for each category. **(B)** Pieplot shows the composition of the merged database. Number of entries in each resource indicated in the parentheses. **(C)** TRA and TRB availability for the six major resources.

To compare currently available TCR-pMHC prediction models, we first collected data from the most comprehensive public resources, including 10X Genomics, McPAS-TCR, VDJdb, ImmuneCODE, TBAdb and IEDB, then preprocessed separately and afterwards merged into one dataset (TCR preprocessed dataset, tpp dataset). The general process is depicted in **Figure 1**. The tpp dataset amounts to 113762 entries, out of which 32237 entries contain paired TCR chains, 7167 entries contain only α chains (TRA) and 74358 entries contain only β chains (TRB)(**Figure 2A**). The composition of the database is shown in **Figure 2B**. From different data resources, ImmuneCODE contains exclusively TRB information, whereas VDJdb contains the highest number of paired chain examples (**Figure 2C**). If we further look into the binding pairs between TCRs and peptides presented by MHC molecules, there is a strong imbalance concerning the peptides, i.e. 0.12% of all peptides (20/1659) account for 58.38% of the total entries (66413/113762). More detailed peptides origin concerning different disease categories for each resource is shown in **Figure S1**.

**Figure 3:**
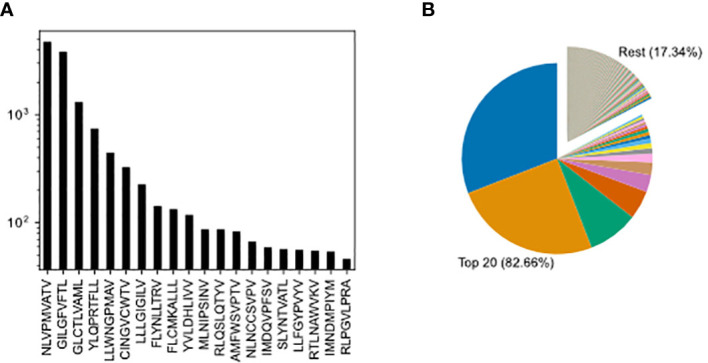
Overview of TCR-pMHC positive binding examples for $d_{base}$ . **(A)** Barplot shows the number of entries for the top 20 peptides. **(B)** Pie chart shows the constitution of examples for the top 20 peptides vs. the rest in $d_{base}$ .

In order to compare the performance of TITAN, NetTCR-2.0, ERGO, DLpTCR and ImRex, they need to be trained and tested on the same data. We constructed a base dataset ( $d_{base}$ ), which fulfills all the requirements from these models so that every model can be trained and tested on it. The criteria are: 1) peptide length equals to 9; 2) CDR3 TRB length in the range of 10 to 18; 3) peptides are presented by the HLA-A*02 MHC allele. After applying these criteria, we removed duplicates based on the CDR3 TRB and peptide, this resulted in a total of 15331 entries for $d_{base}$ , across 15039 CDR3 TRB and 691 peptides. The data in $d_{base}$ is highly imbalanced towards high frequent peptides, 82.66% (12672) of all entries are derived from the top 20 most frequent peptides. The total entries for the top 20 peptides in $d_{base}$ is shown in **Figure 3A**. The imbalance of TCRs pairing with the top 20 peptides is highlighted in **Figure 3B**. The top 20 peptides are paired with 82.66% of the total TCRs while the remaining peptides are paired with the remaining 17.34% TCRs. Furthermore, 517 out of the total 691 peptides have less than five examples per peptide in $d_{base}$ .

### 2.2 Comparison of model performance on d_base_ indicates that current DL models perform similarly well regardless of model complexity

After acquiring the merged dataset and filtering with the most strict requirements of all tested models we obtained the $d_{base}$ dataset. In the creation of $d_{base}$ dataset there were two steps necessary. First, we split the data into five folds as we use 5-fold cross-validation. We used two different splitting methods (see subsection 4.1.2), uniform splitting which keeps the peptide distribution equal across all folds and strict splitting which keeps the peptides unique in each fold. The second prerequisite was to generate negative examples (see subsection 4.1.3), i.e. by assigning combinations of CDR3 β sequences and peptides that do not bind to each other.

Next, we tested six different DL models from five publications. The chosen models predict the binding between a given TCR-pMHC pair. The feature input are the CDR3 TRB sequence of the TCR, and the amino acid (aa) sequence of the peptide. The six models differ in their approaches to embed and process the given features. This subsection compared the different approaches and measured their performance. Models were trained and tested on $d_{base}$ using 5-fold cross-validation. In **Table 1** the tested models are summarized.

**Table 1:** Overview of the tested models.

Models	Architecture	Embedding	Year	Trainable parameters
TITAN Weber et al. (11)	Bimodal attention networks, pretrained with BindingDB.	Encoded peptides with SMILES, TCRs with BLOSUM62 and padded to the same length.	2021	15,506,099
DLpTCR Xu et al. (14)	Ensemble network out of: FCN, CNN and ResNet	depending on subNN: PCA on 500 amino acid indices, one-hot encoded or 20 different physicochemical properties (PCP)	2021	10,454,869
ERGO Springer et al. (13)	Autoencoder or LSTM → Multilayer perceptron (MLP)	One-hot encoded and embedded with either LSTM or Autoencoder	2020	580,299 (Autoencoder) or 6,557,421 (LSTM)
NetTCR2.0 Montemurro et al. (12)	CNN	Both sequences were encoded using the BLOSUM50 matrix	2021	21,345
ImRex Moris et al. (15)	CNN, L2 regularization penalty of 0.01. Dual-input CNN architecture	PCP interaction map between CDR3 and peptide sequence with 20x11x4 dimensions.	2020	248,257

**Figure 4:**
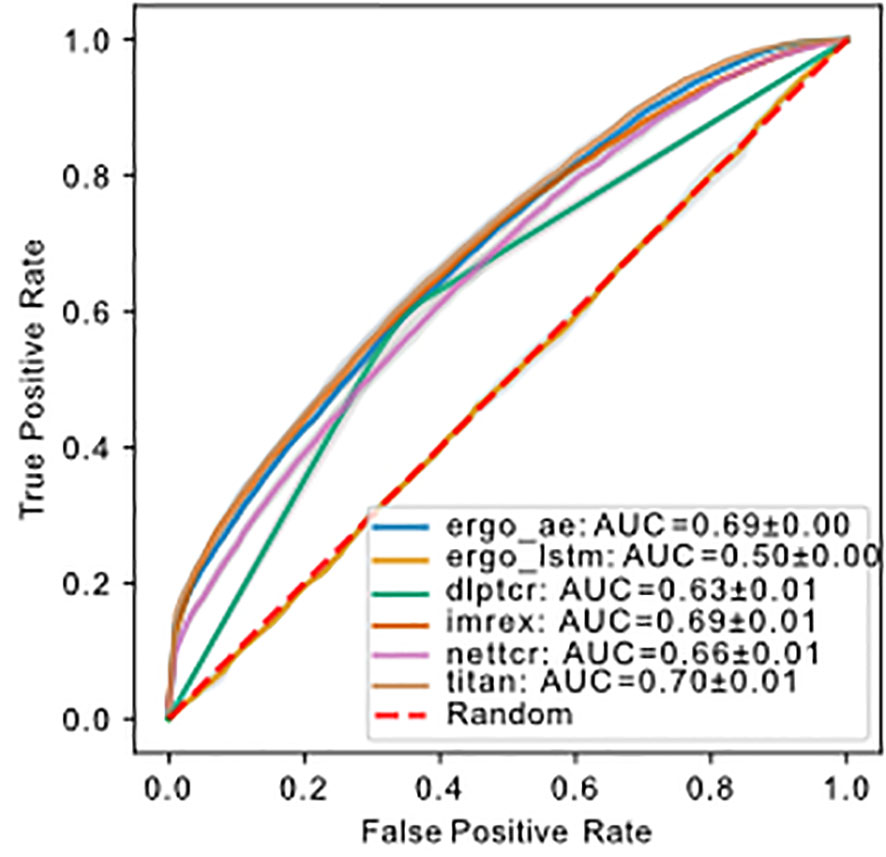
ROC of models predicting binding of TCR-pMHC trained and tested on $d_{base}$ using uniform splitting. The dashed red line indicates performance of random guessing. ROC curve for DLpTCR looks “linear”, because DLpTCR outputs a binary and not a continuous probability.

The number of trainable parameters (**Table 1**) of a model indicates the model complexity. We do not see a correlation between the number of trainable parameters and performance of the model. We used a 1:1 ratio of positive:negative binding examples for both training and testing sets. The ROC-AUC score of each model on $d_{base}$ , except for ERGO with the embedding of long short-term memory (LSTM), were above fifty percent (**Figure 4**). Therefore, almost all models predicted the outcome of a given TCR-pMHC pair better than random guessing. With the exception of ERGO with the LSTM embedding, no ROC-AUC score stood out and performances of those models were within 0.66±0.04 ROC-AUC. A summary to compare the obtained ROC-AUC from the original work and our measurements using a distinct dataset is given by **Table S1**.

### 2.3 Model performance on uniform or strict split data indicates that current models do not perform well on unseen peptides

A generalized prediction model will find interaction patterns that are transferable to new TCR-pMHCs examples. We used two training and testing splitting methods (see subsection 4.1.2) to generate uniform and strict splitting data sets. The main difference of uniform splitting and strict splitting is whether the peptide in the testing set appears in the training set. In uniform splitting the peptides in the testing set also exist in the training set (seen peptides), whereas the peptides in strict splitting have no overlap between training and testing set (unseen peptides). For a generalized TCR-pMHC binding prediction model, it should be able to predict binding on unseen peptides.

**Figure 5:**
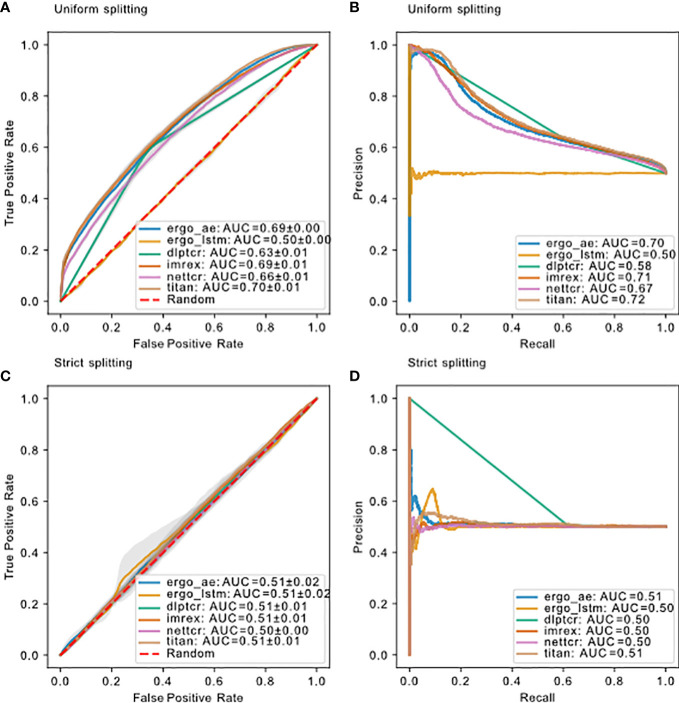
Model performance on d_base_ using different splitting methods. **(A)** ROC curve and **(B)** PR curve for models using uniform splitting. **(C)** ROC curve and **(D)** PR curve for models using strict splitting. The dashed red lines indicate performance of random guessing. ROC and PR curve for DLpTCR looks “linear”, because DLpTCR outputs a binary and not a continuous probability.

The model performance for all models using these two splitting methods is compared in **Figure 5**. DLpTCR returns a binary in its prediction, and this explains why the curves for DLpTCR in **Figure 5** only connect three points. Every other model outputs a value between zero and one, which serves as a probability for the given TCR-pMHC pair to bind. A continuous probability value can generate more points in the ROC and PR curve, if one vary the threshold for a binding and unbinding prediction. Model performance collapsed for strict splitting (comparing **Figures 5A** with **Figure 5C** or **Figure 5B** with **Figure 5D** for each model), indicating that current models do not generalize to unseen peptides.

### 2.4 Collapsing performance on d_bal_ suggests that 5-10 examples per peptide is not sufficient for training state-of-the-art DL models

**Figure 6:**
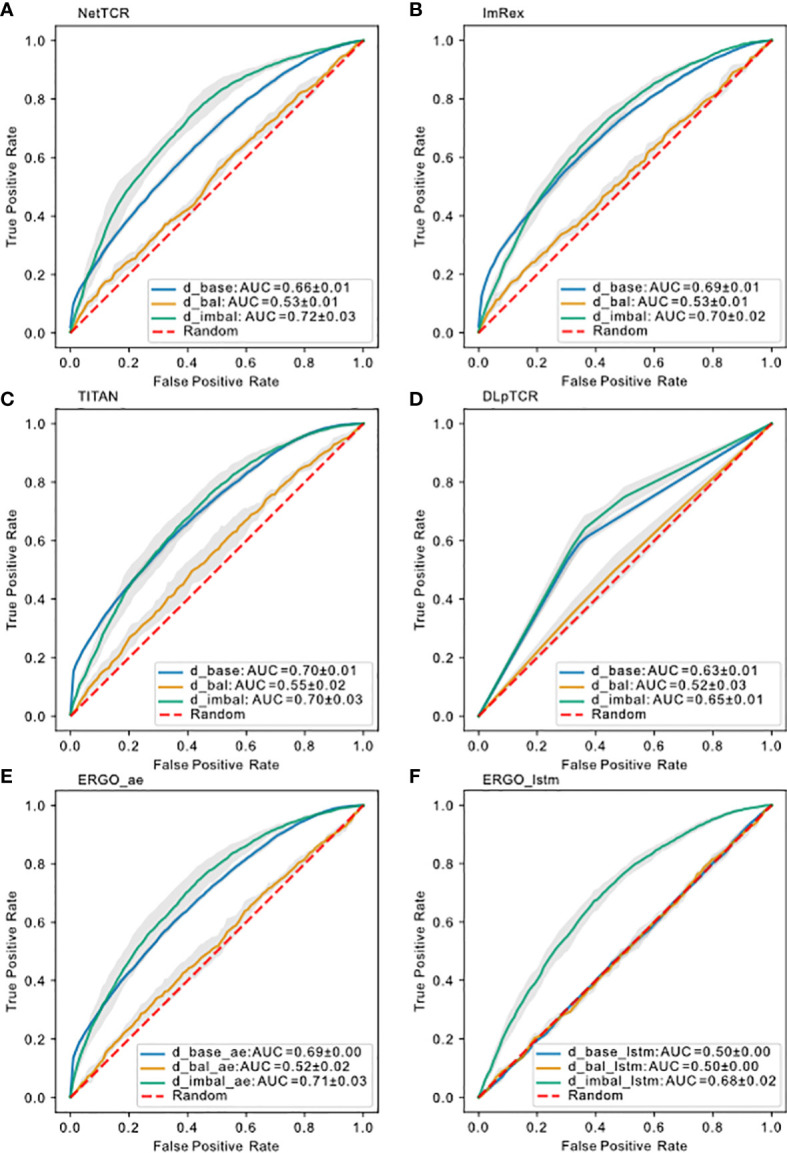
Model performance on different datasets using uniform splitting. ROC curve for **(A)** NetTCR-2.0, **(B)** ImRex, **(C)** TITAN, **(D)** DLpTCR (Curves looks “linear”, because DLpTCR outputs a binary and not a continuous probability), **(E)** ERGO Autoencoder model and **(F)** ERGO LSTM model using $d_{base}$ , $d_{bal}$ and $d_{imbal}$ . The dashed red diagonal line indicates performance for random guessing.

After comparing the results for $d_{base}$ using uniform/strict splitting, we realized that current models are not able to predict the binding for unseen peptides. Since results for uniform splitting showed moderate prediction ability, we suspected that these models learned for the high frequent peptides. In order to elucidate this, we prepared a new balanced data set ( $d_{bal}$ ) to test this hypothesis. Based on $d_{base}$ , we filtered out entries with less than 5 examples per peptide and afterwards we downsampled (see chapter 4.1.4) each unique peptide, so that each peptide in $d_{bal}$ only contains 5-10 examples. This resulted in $d_{bal}$ with a total of 2812 examples, across 1397 unique CDR3 TRB sequences and 174 unique peptides. Training the models on $d_{bal}$ , we saw a complete collapse of performance for the models (**Figure 6**), similar to $d_{base}$ strict splitting. This indicates either that 5-10 examples per peptide is not sufficient for a predictive model to learn the general TCRs-pMHC binding rules or that a total of 2812 examples is not enough to train and test the models on. In the following subsection we investigated how data imbalance impacted the model performance.

### 2.5 Model performance comparison on d_base_ and d_imbal_ indicates that “success” is only due to the most frequent peptide

The difference between $d_{bal}$ and $d_{base}$ is in the size and the imbalance regarding the peptide distribution. The degree of balance can be calculated with the formula for Shannon entropy,


(1) $$Balance=\frac{-1}{\log(K)}\sum_{i=1}^{K}c_{i}log(c_{i})$$

with K as the number of unique peptides and $c_{i}$ as the occurrence in percentage for peptide i . We constructed $d_{imbal}$ to investigate whether the peptide imbalance or the data size impacts the performance more. This dataset included all available data for the most frequent peptide (mfp) (“NLVPMVATV”), but filtered and downsampled the remaining peptides (non-mfp). In total, $d_{imbal}$ has 12268 entries, with 7678 unique CDR3 TRB sequences and 174 unique peptides. This dataset has a higher peptide imbalance than $d_{base}$ and a smaller size (see **Table S2**).

We would expect $d_{base}$ which contains more input data to have a better performance over $d_{imbal}$ if the model can learn a general binding rule. However, models trained on $d_{imbal}$ had a prediction power comparable to models trained on $d_{base}$ , and even slightly better than models trained on $d_{base}$ (**Figure 6**). In the case of ERGO with LSTM embedding, which was as bad as random guessing if trained on $d_{base}$ , if trained on $d_{imbal}$ we saw an increase in prediction performance. Therefore, we conclude that peptide imbalance impacts the performance more than the size of the data. This result also suggests that all models learned the binding rule for the most frequent peptide examples.

### 2.6 Performance increases with peptide imbalance

**Figure 7:**
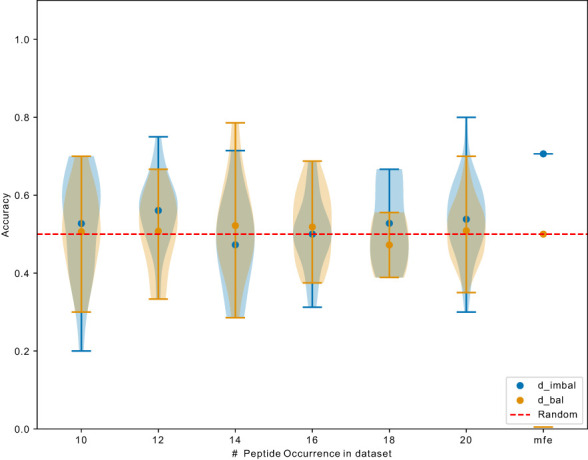
Exemplary comparison of NetTCR-2.0 performance trained on $d_{imbal}$ and $d_{bal}$ . Data points indicate accuracy for models (trained on different datasets) testing on unique peptide with different occurrence. mfp: most frequent peptide 20 examples in $d_{bal}$ and 9476 examples in $d_{imbal}$ .

Next, we investigated whether the learned most frequent peptides from $d_{imbal}$ can be transferred to predict the binding for less frequent peptides. Overall, the ROC-AUC scores for the models trained on $d_{imbal}$ were significantly higher than the one trained on $d_{bal}$ (**Figure 6**). If models trained on $d_{imbal}$ also showed better performance for the non-mfp, compared to models trained on $d_{bal}$ , this would mean that the learned mfp increases the likelihood of generalization. In **Figure 7**, taking NetTCR-2.0 as an example, we compared the accuracy on non-mfp data using models trained on the two datasets and no change in performance was observed. We observed a strong data dependency regarding the performance of all models (**Figure S2**). In retrospect, the success of previously published models could thus be attributed to the peptide imbalance within each dataset.

## 3 Discussion

In this work, we compared different state-of-the-art models for the prediction of TCR-pMHC binding. We chose to use these models as they were supplied, without optimizing them for our datasets. This might have advantages for some models and disadvantages for others, but the aim of this study was to make a consistent comparison across all available data, rather than to compare the peak performance of these models. The data preprocessing and filtering criteria were based on the intersection requirements of all models. In this way we fairly tested the models for their generalization ability using the same input data. By using different train/test splitting methods, we were able to contrast the performance of the models between unseen and seen peptides. Our findings clearly show that all models with different complexity fail to predict on unseen peptide examples. This is consistent with the findings of Grazioli et al. Grazioli et al. (16), who contrasted the performance between uniform and strict splitting as well. They show that ERGO II as well as NetTCR-2.0 performs worse in strict splitting. Here, we have also tested NetTCR-2.0 and a predecessor model of ERGO II (ERGO), but additionally includes TITAN, DLpTCR and ImRex to cover all the current state-of-the-art models (**Table 1**) for TCR-pMHC binding prediction. We showed that the performance stays the same across models with different complexity. Notably, Grazioli et al. suggested that TITAN is a potential candidate to have a generalized prediction prowess. TITAN Weber et al. (11) by Weber et al. applied strict splitting themselves and measured a performance of up to 0.62 ROC-AUC. However, we could not replicate this result based on our dataset. TITAN did not perform significantly better than the other models tested in our study, despite using the most advanced model architecture. A possible explanation why Weber et al. measured better performances could be that they only used data from VDJdb (peptides from various origin) and ImmuneCODE (exclusively COVID data). Merging those two datasets will result in mostly peptides associated with COVID (105/192 [54.69%] assuming VDJdb does not contain many COVID data). Even if the peptides in the testing and training sets are disjoint in strict splitting, there might be similar peptides across the training and testing set, due to their same origin from COVID. This may have contributed to the better performance reported. If this hypothesis is true, given enough training examples, it might be possible for TITAN and other models to not only predict peptide-specific binding but also origin-specific binding. Based on current available data, models work better for epitope specific predictions, not for general predictions.

We also investigate the impact of peptide imbalance on the performance of the models. To the best of our knowledge, we have not seen similar training and testing of the models on different data scenarios ( $d_{base}$ , $d_{bal}$ and $d_{imbal}$ ). The data scenarios vary in size and peptide distribution. We suggest that peptide imbalance contributes more to a better performance of the models than size, a finding that was also made in antibody-antigen prediction Robert et al. (17). It will be interesting to see whether the models perform well purely because of peptide frequency, or whether other factors such as biological or physicochemical properties may influence performance. This can be explored by clustering peptides based on physicochemical features using different approaches (HMM Rabiner (18) to KNN Taunk et al. (19), and checking the performance. With various clustering methods to choose and an abundant set of parameters, we would continue our research on this in the future.

This is consistent with the consensus that currently available data are not sufficient, an issue raised so far by every study of these models Weber et al. (11); Montemurro et al. (12); Springer et al. (13); Xu et al. (14); Moris et al. (15). The way we prove data dependence in this study may not take into account the effects of sequence features or similarities, but this actually strengthens the findings. We have shown in the most straightforward and transparent way that down to the smallest granularity (peptide as a categorical variable), data imbalance has a major impact on performance. Our results support the idea that a generalized predictive model requires data that is not only large but also massively diverse to uncover a large range of potential pMHC-TCR binding rules. A suggestion would be to specifically increase the screening for scarce peptides to further increase dataset diversity. TCR sequencing on the single cell level is a rapidly progressing field, so affordable screening technology to do so with high fidelity should be available soon.

The hypothesis that models such as TITAN might be able to predict unseen but similar peptides or peptides from the same origin is a very interesting research question for future work. If this hypothesis holds, we need a global effort to experimentally screen a set of peptides to cover a diverse peptides pool, and make use of the generated data for constructing a generalizable prediction model.

A limitation of this study is that our datasets only comprised TCRs from CD8^+^ T cells pairing with peptides presented by the HLA-A*02 allele without considering other MHC alleles, however, it was important to exclude additional variables such as HLA isotypes at this point. Moreover, we only compared DL models for predicting binding between random TCRs and random pMHC, not epitope-specific models (i.e. the prediction of whether random TCRs bind to a specific peptide). Meysman et al. have compared superficially different approaches to TCR-pMHC binding Meysman et al. (20), but also raised the importance of a truly independent benchmark. They reveal that additional information like CDR1/2 improved the prediction, but they did not investigate the role that imbalance, size or overtraining might have on model performance by using those additional features within the used dataset.

## 4 Methods

### 4.1 Data preprocessing

#### 4.1.1 Date merging and preprocessing

We downloaded the data from six different resources. We unified the column names of (CDR3 TRA, CDR3 TRB, peptide and MHC, etc.). We only kept entries that have a peptide and at least either a CDR3 TRA or TRB sequence. Only TCRs sequences and peptide sequences that use the 20 valid amino acid residues are kept. After this quality control, all data from different resources were merged into one dataset (tpp dataset), duplicates in this merged dataset were then removed. The preprocessing of the merged dataset and prefiltering for different datasets are shown in **Figure 1**.

#### 4.1.2 Splitting

We explored two different splitting methods (**Figure S3**). The first method kept the distribution of the peptide in each part (uniform splitting). The second method distributed peptides to each part, so that no peptide is in two different parts (strict splitting). The strict splitting we used here is inspired by the splitting method from the TITAN [20] model. Strict splitting was only used for $d_{base}$ (**Figure 1**). $d_{base,strict}$ and $d_{base,uniform}$ vary in size (**Table S2**), because strict splitting includes peptides with less than five examples. In subsection 2.1 we showed a data imbalance in peptides. For the 5-fold cross-validation in strict splitting we ensured, that each fold did not have a peptide exceeding more than half of its entries. If a peptide has more entries it will be downsampled to the half of the fold size. Uniform splitting exclude peptides with less than five examples, because uniform splitting requires at least one example for each peptide in all five folds. **Table S2** shows that $d_{base,strict}$ have more unique peptides but less total entries compare to $d_{base,uniform}$ . In $d_{base,strict}$ , we downsampled many positive examples (for high frequent peptides) in order to generate negative examples within each fold without external reference TCR repertoire, this reduces the total number of examples in the dataset, while in $d_{base,uniform}$ , some examples for less frequent peptides were filtered out to ensure at least one example in each fold.

#### 4.1.3 Negative example generation

The collected and merged dataset only have positive binding examples. The training of neural network models for binding prediction requires positive and negative examples. The negative examples were created by rearranging TCR-pMHC pairs. Let $T_{a,0}$ , $T_{a,1}$ be T cells which bind to peptide $p_{a}$ and $T_{b,0}$ , $T_{c,0}$ bind to $p_{b}$ or $p_{c}$ respectively. By pairing $T_{a,0}$ , $T_{a,1}$ with $p_{b}$ and $p_{c}$ we created negative pairing examples. Statistically it is unlikely for the new generated TCR-pMHC pair to bind. This generation of negative examples agrees with most models original work. For each positive example a negative example was created. $d_{base}$ , $d_{bal}$ and $d_{imbal}$ have therefore a positive to negative ratio of 1:1 . In case one peptide needs more $T_{i}$ (i.e. $d_{imbal}$ ) to generate the same amount of negative examples, $T_{i}$ from previous downsampling served as additional reference $T_{i}$ .

#### 4.1.4 Downsampling

Peptides are not uniformly distributed throughout tpp dataset. Some peptides occur only a few times (low frequent peptides) and some occur hundreds of times (high frequent peptide). For $d_{bal}$ and $d_{imbal}$ we downsampled the high frequent peptides to keep only 10 random examples for each peptide.

### 4.2 Model performance measurement

We downloaded the source code for all models from their respected GitHub repository. We evaluated all models with 5-fold cross-validation. We used our datasets to train the models with the default parameters. The performance is measured by the area under the receiver and operator curve (ROC-AUC) Davis and Goadrich (21), as well as the area under the precision recall curve (PR-AUC)Saito and Rehmsmeier (22). The best ROC-AUC models was saved and evaluated on testing set.

## Data availability statement

The original contributions presented in the study are included in the article/**Supplementary Material**. Further inquiries can be directed to the corresponding authors.

## Author contributions

LD and CL contributed equally to this work and share first authorship. IP and SB contributed to conception and design of the study. LD and CL collected and preprocessed the data. SA supported LD, and CL in performing the comparison of the existing prediction tools, LD and CL interpreted the comparison result. YZ supported in the discussion of this study. LD and CL wrote the draft of the manuscript. All authors contributed to the article and approved the submitted version.
