# Supplementary material for: Performance comparison of TCR-pMHC prediction tools reveals a strong data dependency
Source: Front Immunol. 2023 Apr 18;14:1128326. doi: 10.3389/fimmu.2023.1128326 (PMC10152969; doi:10.3389/fimmu.2023.1128326)
Supplement: Supplementary file 1 [file DataSheet_1.pdf]

7 SUPPLEMENTARY INFORMATION

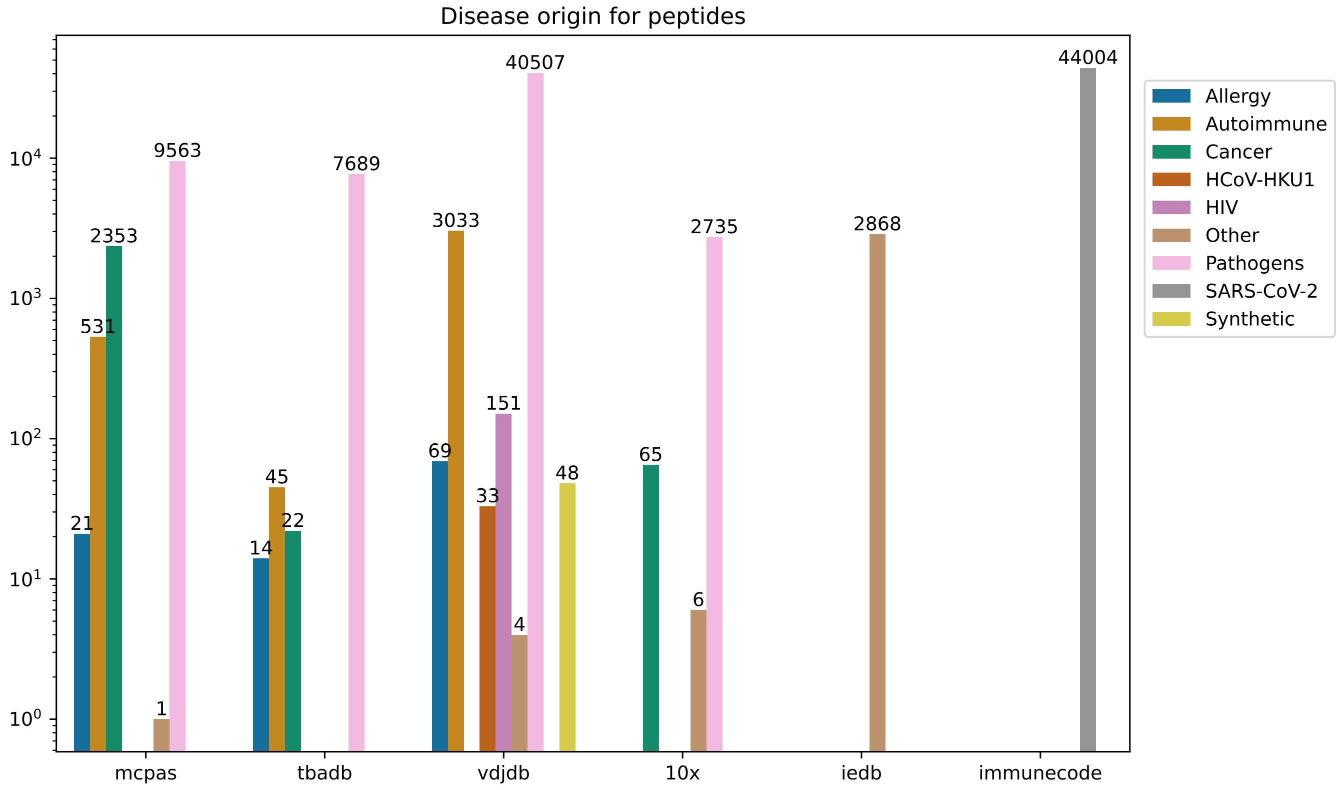

Figure S1. Disease origin for peptides. Peptides origin for the six major resources based on the related disease. Numbers in the plot show the corresponding records for disease origin in each resource.

Table S1. Peak model performance ROC-AUC in the original work and based on $d_{base,uniform}$.

model	peak original ROC-AUC	d_{base} ROC-AUC
TITAN	0.87 ± 0.01	0.70 ± 0.01
NetTCR-2.0	0.80	0.66 ± 0.01
ERGO AE	0.958	0.69 ± 0.00
ERGO LSTM	0.970	0.50 ± 0.00
DLpTCR	0.91	0.63 ± 0.01
ImRex	0.68 ± 0.01	0.69 ± 0.01

Table S2. Overview of constructed datasets.

	$d_{base,strict}$	$d_{base,uniform}$	d_{bal}	d_{imbal}
# entries	15964	28716	2812	12268
# unique peptide	691	174	174	174
# unique CDR3 Beta	7805	14141	1397	7678
Shannon entropy	0.65	0.49	0.99	0.33

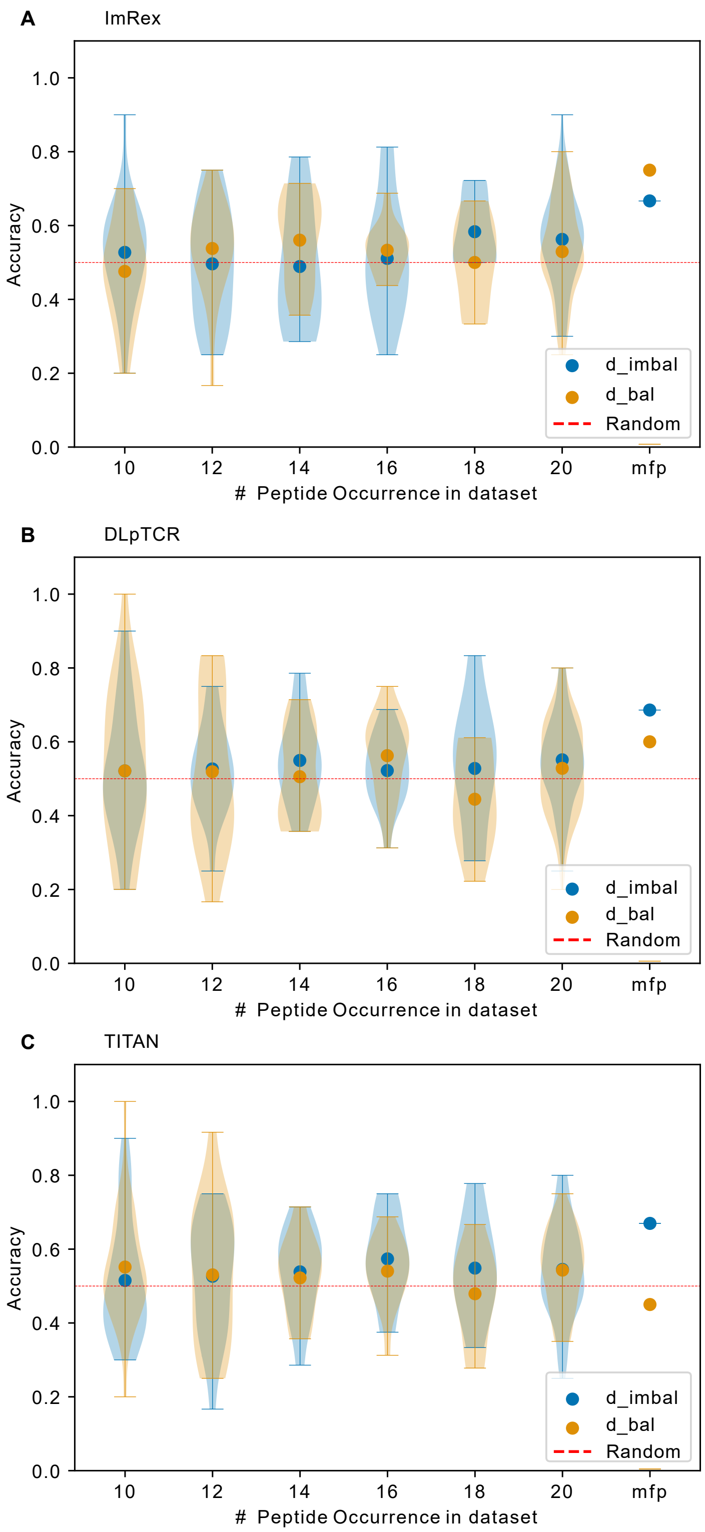

Figure S2. Performance trained on d_{imbal} and d_{bal} for A) ImRex, B) DLpTCR and C) TITAN. Data points indicate accuracy for models (trained on different datasets) testing on unique peptide with different occurrence. mfp: most frequent peptide 20 examples in d_{bal} and 9476 examples in d_{imbal}.

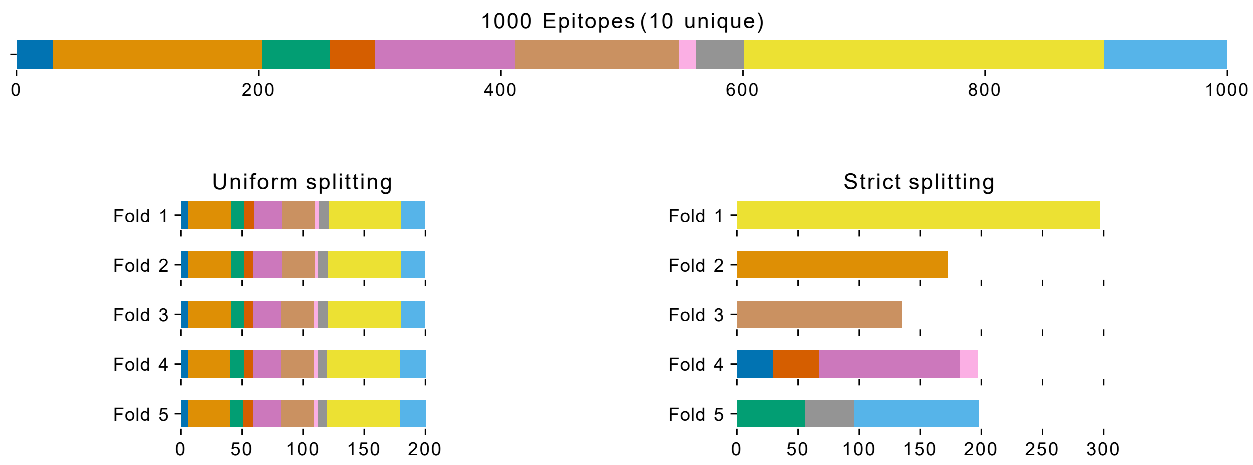

Figure S3. Uniform and strict splitting schematically demonstrated with an imbalanced dataset of 1000 entries and 10 unique peptides.
